# Exploratory factor analysis for differentiating sensory and mechanical variables related to muscle-tendon unit elongation

**DOI:** 10.1590/bjpt-rbf.2014.0152

**Published:** 2016-03-22

**Authors:** Mauro H. Chagas, Fabrício A. Magalhães, Gustavo H. C. Peixoto, Beatriz M. Pereira, André G. P. Andrade, Hans-Joachim K. Menzel

**Affiliations:** 1 Escola de Educação Física, Fisioterapia e Terapia Ocupacional, Universidade Federal de Minas Gerais (UFMG), Belo Horizonte, MG, Brazil

**Keywords:** multivariate analysis, biomechanical properties, hamstring muscles, stretching exercises, stretch tolerance, movement

## Abstract

**Background:**

Stretching exercises are able to promote adaptations in the muscle-tendon unit (MTU), which can be tested through physiological and biomechanical variables. Identifying the key variables in MTU adaptations is crucial to improvements in training.

**Objective:**

To perform an exploratory factor analysis (EFA) involving the variables often used to evaluate the response of the MTU to stretching exercises.

**Method:**

Maximum joint range of motion (ROM_MAX_), ROM at first sensation of stretching (FST_ROM_), peak torque (torque_MAX_), passive stiffness, normalized stiffness, passive energy, and normalized energy were investigated in 36 participants during passive knee extension on an isokinetic dynamometer. Stiffness and energy values were normalized by the muscle cross-sectional area and their passive mode assured by monitoring the EMG activity.

**Results:**

EFA revealed two major factors that explained 89.68% of the total variance: 53.13% was explained by the variables torque_MAX_, passive stiffness, normalized stiffness, passive energy, and normalized energy, whereas the remaining 36.55% was explained by the variables ROM_MAX_ and FST_ROM_.

**Conclusion:**

This result supports the literature wherein two main hypotheses (mechanical and sensory theories) have been suggested to describe the adaptations of the MTU to stretching exercises. Contrary to some studies, in the present investigation torque_MAX_ was significantly correlated with the variables of the mechanical theory rather than those of the sensory theory. Therefore, a new approach was proposed to explain the behavior of the torque_MAX_ during stretching exercises.

## Bullet point

- Stretching exercises is routinely used in both rehabilitative and sports areas.
- Two main theories (mechanical and sensory) have been suggested in the literature.
- The present EFA revealed two major factors solidary to these two theories.
- A *continuum* of individual tolerance was verified through the sensory variables.
- Passive torque was associated with mechanical variables instead of sensory variables.

## Introduction

Limited flexibility may predispose active people to musculoskeletal injuries^1^, as shortened muscles have been associated with the incidence of lower limb injuries^2^. Consequently, stretching is an intervention routinely incorporated into rehabilitation programs, recreational sports, and physical activities. However, the importance of stretching exercises in preventing injuries is not well defined^3^. Indeed, complex methodological procedures are required to better understand the effects of stretching. Toft et al.^4^ noted the need to investigate the effects of stretching not only through the joint range of motion (ROM) recordings but also through resistive torque.

Traditionally, joint ROM has been the main variable analyzed in studies on the response of muscle-tendon unit (MTU) to stretching exercises in humans *in vivo*. However, Weppler and Magnusson^5^ suggested a multidimensional investigation of this response, in which resistive force to stretching, muscle cross-sectional area (CSA), and time should be considered dimensions in addition to the MTU length (often represented by the single variable joint ROM). Studies which considered more than one dimension, such as peak torque, passive stiffness (ratio between varying passive torque and varying ROM), and energy (area under the curve passive torque vs. ROM)^6^ ^,^ ^7^, have provided additional information in the understanding on the MTU response to stretching.

Another variable which has been investigated in recent studies was initially introduced by Halbertsma and Goeken^6^, which called it “first sensation of pain” and operationally defined it as the ROM value registered at the time of the “first sensation of pain”. However, these authors did not instruct volunteers to reach any pain level. They actually measured the first sensation of stretch, since they defined “pain” as the first feeling of muscle tension during passive stretching.

Additional understanding of the MTU response to stretching can be obtained by increasing the number of both mechanical and sensory variables. In this context, assessing these variables may allow researchers to argue for or against a particular theory used to explain the MTU response to stretching^8^. Aiming to expand the analysis, other studies investigated the relationships between different variables^9^ ^-^ ^11^. These studies found statistically significant correlations between ROM and passive stiffness, but their results differed in magnitude and direction. Aquino et al.^10^ and Kubo et al.^11^ found negative correlations between ROM and passive stiffness (r=-0.78 and r=-0.48, respectively), whereas Blackburn et al.^9^ found a positive correlation (r=0.52). This difference in direction is due to the operational definition of flexibility used: while Blackburn et al.^9^ defined the initial position as the knee full extension, Aquino et al.^10^ and Kubo et al.^11^ considered the joint extension as the final position.

However, the muscle stiffness measurements in those studies were not normalized by muscle CSA. This is an important procedure because Chleboun et al.^12^ demonstrated a high correlation between CSA and passive stiffness (r=0.92). Recently, Blazevich et al.^13^ found a positive correlation between passive peak torque (usually strongly correlated to CSA) and maximum joint ROM (r=0.69, P<0.001). Nonetheless, there is a lack of information regarding how each of these variables and their interrelationships determine the behavior of MTUs during stretching.

Considering the increasing number of the previously reported variables as well as the interrelationships among them, a study verifying how these variables are related within a multivariate analysis context may enhance the understanding on the theories behind the MTU’s adaptations after stretching exercises. The application of exploratory factor analysis (EFA) can be useful because it permits the grouping of variables into smaller sets of factors. Nevertheless, to the best of our knowledge, the existence of different factors related to the MTU response to stretching and the respective sets of variables that characterize these responses have not yet been reported in the literature. Consequently, the aim of this study was to investigate the factors identified through an EFA of the most common variables related to the MTU stretching maneuver. This type of analysis can indicate whether the variables represent distinct characteristics of the MTU response and can facilitate the understanding of the sensory and mechanical mechanisms. Therefore, the purpose of this study was to improve the understanding of the MTU response to stretching and consequently lead to better interventions to improve ROM in both rehabilitative and sportive areas.

## Method

### Participants

Based on the findings of a pilot study, the sample size calculation was performed by using the equation $x\pm \frac{t*s}{\sqrt{n}}$, where *x* was the mean value of the variable with higher coefficient of variation (e.g. stiffness), *t* was 1.96 (considering the probability of a Type-I error equal to 0.05), *s* was the standard deviation, and *n* was the sample size for each experimental group. By means of *x*=0.9 and *s*=0.4, the sample size calculation indicated an *n* equal to 34 participants. Due to the possibility of losing participants during testing, 36 healthy young participants (18 males and 18 females; mean±SD: age 24.2±3.2 years; height 169.8±7.9 cm; and body mass 67.2±12.9 Kg) were recruited to participate in the study.

The participants were free of any health condition that could impair the completion of the tests such as pain or recent lower limb injury. Participants were excluded if they presented a knee extension ROM higher than 135° during the familiarization session, routinely performed any stretching or strength training, or experienced any disabling condition during the tests. Both lower limbs were considered for analysis, resulting in 72 sampling units. This study was approved by the Research Ethics Committee (approval no. ETIC 246/08) of Universidade Federal de Minas Gerais (UFMG), Belo Horizonte, MG, Brazil.

### Data collection and analysis

Each participant completed three sessions of data collection and underwent to MRI (magnetic resonance imaging) scan. In the first session, they received information about the study and signed an informed consent form. Anthropometric measurements were taken, followed by familiarization trials with an isokinetic dynamometer (Flexmachine)^14^. Subsequently, two sessions of data collection were conducted with a 24-48 h interval between sessions. Data collected in the second session were used for the EFA, whereas those collected in the third session were used for the reliability calculations. Finally, between the data collection sessions and the MRI, a maximum 15-day interval was allowed. It was expected that the participants’ muscle CSA would not change significantly during this time because, as previously mentioned, the participants did not perform any kind of physical activity.

**Figure 1:**
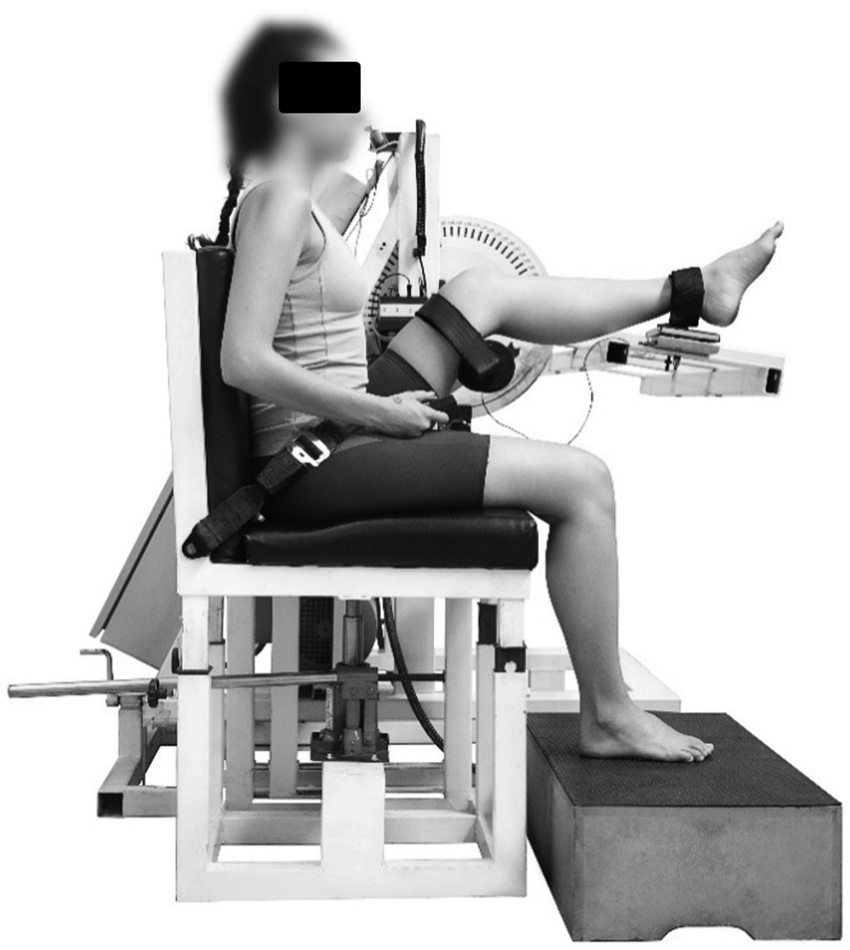
Lateral view of the Flexmachine. This isokinetic dynamometer consists of two chairs connected to a mechanical arm containing a force plate (Refitronic®, Schmitten, Germany). ROM_MAX_, first sensation of stretching, and torque_MAX_ were recorded in the Flexmachine.

During the second session, each participant sat in the dynamometer with thighs and pelvis strapped firmly to the seat. The thigh is positioned at 45° to the ground and the heel on the force plate (Figure 1). During the stretching maneuver, the participants were instructed to achieve the highest ROM (ROM_MAX_) without offering voluntary resistance to the dynamometer’s mechanical arm, and immediately return to the starting position. Passive peak torque (torque_MAX_) and ROM at the first sensation of stretching (FST_ROM_) were registered. Three hamstring muscle stretching maneuvers were performed for each leg, and their average values were taken for analysis.

Since the hamstring muscles (biceps femoris, semitendinosus, and semimembranosus) cross the hip and knee joints, the trunk and thigh were positioned in a way to prevent the participants reaching a complete knee extension^15^. Thus, the resistive force to elongation during the stretching maneuver was due primarily to MTU elongation without involvement of posterior capsular constraints at the knee.

Electromyographic (EMG) activity was recorded by active bipolar Ag/AgCl surface electrodes with a 2 cm inter-electrode distance. Two electrodes were placed over the semitendinosus muscle according to the recommendations of McHugh et al.^16^. The skin was previously shaved and cleansed. EMG data were collected at 1 KHz and filtered with a second-order high-pass Butterworth filter of 15 Hz. All devices were connected to the computer through a Data Translation analog/digital converter (DT BNC USB Box 9800 Series). Collection and analysis of the signals were performed using the software DasyLab 9.0 (Data Acquisition System Laboratory, DasyTec, Amherst, NH, USA).

**Figure 2:**
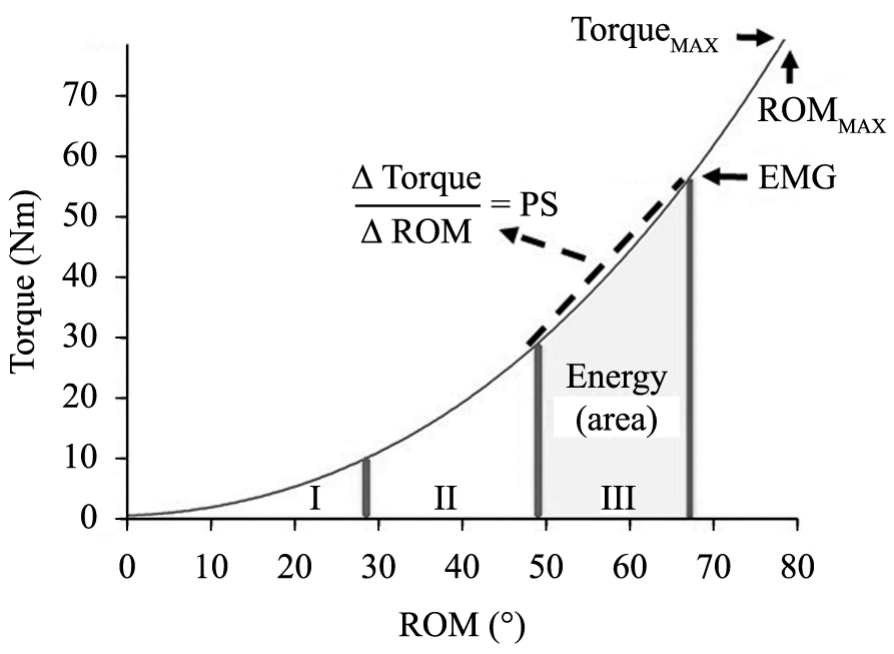
Schematic representation of the torque (N.m) vs. ROM (°) curve. Torque: resistive force to stretching; ROM: joint range of motion; I, II, III: first, second, and third part of the curve; PS: passive stiffness, defined as the ratio between the varying torque and varying ROM in the third part of the curve; EMG: cutoff point of the curve defined by a significant increase in EMG activity; ROM_MAX:_ maximum range of motion; Torque_MAX:_ peak torque.

For the passive stiffness and passive energy calculations, the influence of EMG activity on torque and ROM measurements was minimized by a cutoff criterion. Torque (N.m) and ROM (°) values above the baseline EMG activity, which was the average value of the EMG raw signal amplitude (mV) during the first 2s of stretching plus two standard deviations, were disregarded. In sequence, the curve passive torque vs. ROM was plotted and divided into thirds (Figure 2). Passive stiffness (N.m/°) was the slope of the last third of the curve^9^ ^,^ ^17^, and passive energy (J) was the area under the last third of the curve. Usually, passive stiffness and energy are calculated in the last third of the curve passive torque vs. ROM because the coefficient of variation is lower (6-15%) than that of the first third (20–28%) according to Magnusson^15^.

The normalization of the passive stiffness and passive energy was performed by dividing the passive torque by the hamstring muscle CSA (cm^2^) of each participant. Then, the passive stress (N.m/cm²) *vs.* ROM (°) curve was plotted, and both normalized stiffness (N.m.cm^−2^/°) and normalized energy (J/cm²) were calculated in the same manner as passive stiffness and passive energy. The transverse plane of both thighs were MRI-scanned at the distal third of the femur and used for the CSA calculation using the *GE Signa* 1.5 Tesla system (General Electric, Milwaukee, WI, USA), T1-weighted; repetition time/echo time, 300 ms/12 ms; 256 × 256 matrix size; 400-mm field of view; 10-mm slice thickness; and a 1-mm interval between slices.

### Statistical analysis

EFA is a collection of methods for explaining the correlations among variables. It aims to identify a smaller number of new alternatives and non-intercorrelated variables (called factors) that summarize key information from the original variables. The following assumptions recommended by Hair et al.^18^ were verified before performing EFA: 1) the structure of correlation among the variables through both Kaiser-Meyer-Olkin (KMO) statistics and Bartlett’s sphericity; 2) retention of a minimum number of factors with an eigenvalue greater than one via the Kaiser-Guttman criterion; 3) factorial rotation by the varimax method; and 4) data distribution through Royston’s multivariate normality test. Finally, to increase the robustness and determine the correct number of factors, bootstrap resampling was used since factor loadings are not unique in EFA and different rotation methods can be conducted in EFA to achieve interpretability and identification (e.g. orthogonal and oblique rotation). The analyses were performed using the statistical packages SPSS (version 18.0) and R (version 2011).

## Results

Descriptive and reliability values for CSA, ROM_MAX_, FST_ROM_, torque_MAX_, passive stiffness, passive energy, normalized stiffness, and normalized energy are presented in Table 1.

**Table 1:** Descriptive and reliability data.

**Variables**	**Mean±SD**	**Range**	**ICC_3,1_**	**SEM**	**SEM (%)**
**CSA (cm^2^)**	27.5±7.6	18.3-39.4	0.98	0.59	2.1
**ROM_MAX_ (°)**	94.2±19.7	55.7-131.0	0.99	2.3	2.4
**FST_ROM_ (°)**	74.7±20.0	45.0-117.0	0.99	2.8	3.7
**Torque_MAX_ (N.m)**	37.2±15.5	13.0-77.0	0.98	4.0	10.7
**Passive stiffness (N.m/°)**	0.6±0.3	0.1-1.3	0.96	0.14	23.3
**Passive energy (J)**	1231.6±594.7	342.0-2686.0	0.97	46.3	3.8
**Normalized stiffness (N.m.cm^–2^/°)**	0.02±0.01	0.01-0.05	0.97	0.01	50.0
**Normalized energy (J/cm^2^)**	45.8±21.3	9.7-109.2	0.97	0.07	0.2

CSA: muscle cross-sectional area; ROM_MAX_: maximum joint range of motion; FST_ROM_: ROM corresponding to first sensation of stretching; Torque_MAX_: peak torque; SD: standard deviation; Range: maximum and minimum values; ICC_3,1_: intra-class coefficient of correlation; SEM: standard error of measurement; SEM (%): percentage of SEM to respective mean.

Several tests should be performed to assess the suitability of the database prior to extracting factors in EFA. KMO statistics (measurement of sampling adequacy) and Bartlett’s test of sphericity (to test the hypothesis that the correlation matrix is an identity matrix) are tests to ensure that there is no correlation between the data. According to Thompson^19^, the correct application of EFA could be verified by the results of the KMO statistic (0.715) and the chi-square for Bartlett’s test (776.7, p=0.001). In both cases, the test statistic suggests that the data are adequate to factorial analysis. Moreover, the Royston’s multivariate normality test did not indicate significant deviations from normality (p=0.74), which corroborated the use of EFA. The next step was establishing the number of factors to be extracted. Based on the retention factors with eigenvalues exceeding one^18^, two major factors that explained 89.68% of the total variance were retained; in total, factors 1 and 2 explained 53.13% and 36.55% of the variance, respectively. The mean eigenvalues for each factor and the percentage of variance explained after the varimax rotation are presented in Table 2. The varimax rotation aimed to facilitate the visualization of the relationship between the observed variables and the extracted components given by the factor loadings. Thus, the varimax rotation attempts to maximize the spread of the loadings within factors across variables. In other words, high factor loadings after extraction are further amplified while low loadings are further suppressed.

**Table 2:** Exploratory factor analysis.

	**Eigenvalues (1000 resampling)**			
**Components**	**Mean**	**Standard error**	**Percentage of variance**	**Cumulative percentage**
**1**	3.72	0.17	53.13	53.13
**2**	2.56	0.13	36.55	89.68
**3**	0.41	0.06	5.84	95.52
**4**	0.21	0.05	3.00	98.52
**5**	0.08	0.02	1.08	99.60
**6**	0.02	0.008	0.30	99.90
**7**	0.01	0.007	0.10	100.00

Values referring to the Exploratory Factor Analysis through the principal components extraction method, varimax rotation, and bootstrap technique.

As described by Hair et al.^18^, only variables with factor loadings equal to or higher than 0.65 are considered significant for a sample size equal to 70 observations, with statistical power of 80% and significance level of P<0.05. Table 3 presents the variables factor loadings correlated with factors 1 and 2. Factor loadings are measures of the correlation between the individual variable and the overall factor and determining which variable correlates significantly with a factor cannot be determined a priori. It was found that the variables torque_MAX_, passive stiffness, normalized stiffness, passive energy, and normalized energy predominantly loaded into Factor 1, whereas ROM_MAX_ and FST_ROM_ predominantly loaded into Factor 2. An interpretation of what each factor represents is addressed in the discussion.

**Table 3:** Factor loadings.

**Variables**	**Factors**	
**1**	**2**
**ROM_MAX_ (º)**	0.07	**0.97**
**FST_ROM_ (º)**	–0.12	**0.94**
**Torque_MAX_ (N.m)**	**0.95**	0.21
**Passive stiffness (N.m/°)**	**0.88**	–0.33
**Passive energy (J)**	**0.82**	0.52
**Normalized stiffness (N.m.cm^-2^/º)**	**0.83**	–0.36
**Normalized energy (J/cm^2^)**	**0.72**	0.56

ROM_MAX_: maximum joint range of motion; Torque_MAX_: peak torque; FST_ROM_: ROM corresponding to first sensation of stretching.

## Discussion

The aim of this study was to improve the understanding of the MTU response to stretching. The EFA revealed two major factors with eigenvalues greater than one that explained 89.68% of the total variance. In total, Factor 1 explained 53.13% of the variance, with 36.55% of the variance explained by Factor 2 (Table 2). Five variables (torque_MAX_, passive stiffness, normalized stiffness, passive energy, and normalized energy) were loaded into Factor 1, whereas the other two variables were loaded into Factor 2 (ROM_MAX_ and FST_ROM_).

Torque_MAX_, passive stiffness, normalized stiffness, passive energy, and normalized energy represent a characteristic underlying the investigated data. Excluding torque_MAX_, all other variables grouped into Factor 1 were determined by a cutoff criterion, e.g. a significant increase in EMG activity. Therefore, torque (resistive force to elongation during the stretching maneuver) may be considered passive because the biological tissue deformation happened with minimal participation of voluntary or reflex muscle contractions^20^. Stiffness is the resistive force estimation of MTU in response to changes in its length^9^, whereas energy is the biological tissues’ ability to absorb work that can either be reused in subsequent movements or dissipated as heat^21^. Several studies have used these variables (either CSA-normalized or not normalized) to obtain information about the mechanical properties of MTUs^21^ ^,^ ^22^. Since they presented high correlation coefficients with Factor 1 (r=0.72–0.88; Table 3), this factor can be interpreted as “mechanical.” Similarly, torque_MAX_ also displayed high correlation with the factor “mechanical” (r=0.95).

During stretching maneuvers, different synergist muscles, connective tissues, and articular structures contribute to the torque^23^. However, differentiating the level of participation of each of these structures as well as other mechanisms (like neural influences) for torque_MAX_ remains a challenge^8^ ^,^ ^24^. Johns and Wright^25^ investigated the relative importance of the different tissue types to the joint resistive torque and reported that approximately 41% of the passive torque can be attributed to the muscles and surrounding structures such as tendons, ligaments, fasciae, and joint capsules. Reinforcing this point, Magnusson et al.^17^ presented different values of torque_MAX_ achieved with no significant EMG activity during stretching. In this manner, the mechanical component would be a key point compared to the neural mechanisms. Consequently, it is understandable why torque_MAX_ was correlated with the factor “mechanical”. Nonetheless, aspects influencing torque_MAX_ and ROM_MAX_ may not be the same given that they did not load into the same factor. Since the relative contribution of different structures to the joint resistive torque is still unknown and might be joint-specific, the findings of the present study cannot be generalized to other joints.

The variables ROM_MAX_ and FST_ROM_ were loaded into factor 2. These variables are interrelated and represent a common aspect associated with the stretching tolerance according to the operational definition adopted. Along the stretching maneuver in the Flexmachine, FST_ROM_ corresponded to the individual onset of muscle tension, while ROM_MAX_ (representative measure of muscle extensibility *in vivo*) was determined by the maximum individual tolerance to stretching. A similar procedure was used by Ylinen et al.^26^ and Cabido et al.^14^. Therefore, these variables may represent points pertaining to the ends of a *continuum* of individual tolerance to stretching exercises, where FST_ROM_ and ROM_MAX_ would be the initial and final measures of the individual tolerance, respectively. The foundation for a *continuum* of individual tolerance lies in the relationship found between these variables. Several studies suggested that an increase in ROM_MAX_ after stretching exercises was accompanied by a corresponding increase in FST_ROM_ ^14^ ^,^ ^26^. Through an analysis of the data presented by Halbertsma and Goeken^6^, a high correlation between FST_ROM_ and ROM_MAX_ can be observed (r=0.94, r^2^=0.88, P=0.001). These findings support the existence of a *continuum* of individual tolerance. However, further research is needed to confirm this hypothesis as well as to establish the relationship between ROM_MAX_ and FST_ROM_ by means of regression analysis.

ROM_MAX_ and FST_ROM_ explained 36.55% of the total variance. Since these measures are related to individual tolerance to stretching, a change in MTU response could represent a modulation of individual sensation to stretching. Several studies have suggested that an increase in ROM_MAX_ after conducting acute^7^ ^,^ ^22^ or chronic^7^ ^,^ ^21^ ^,^ ^27^ stretching protocols occurs because of changes in the individual sensation of stretching. In these studies, increases in ROM_MAX_ were accompanied by increases in torque_MAX_ with no significant variation in passive stiffness and energy. Based on that, Weppler and Magnusson^5^ proposed the sensory theory to explain the MTU response to stretching, more specifically the increase in ROM_MAX_, although this explanation has not been universally accepted^28^ ^,^ ^29^. According to the sensory theory, ROM_MAX_ and FST_ROM_ can adequately represent possible changes in the individual sensation of stretching (Factor “sensory”). Consequently, ROM_MAX_ and FST_ROM_ can be used as dependent variables in studies to indicate an alteration in the individual sensation of stretching. Nonetheless, the mechanisms and structures involved in the modulation of the individual sensation of stretching are not yet fully established. Nociceptive nerve endings in muscle and peri-articular tissues may play an important role in this phenomenon^30^ *.*

The new approach described in the present study recommends the use of torque_MAX_ to explain alterations in the MTU response to stretching exercises. Different researchers observed increases in ROM_MAX_ and torque_MAX_ after stretching exercises with no significant change in the variables associated with the MTU’s mechanical properties in both acute^7^ and chronic studies^27^. Higher torque_MAX_ values have been linked to an increased stretch tolerance (e.g. alteration in the individual sensation of stretching). Hence, participants tolerating larger torque_MAX_ values display greater ability to achieve higher ROM_MAX_ values, which indicates a cause-and-effect relationship. Blazevich et al.^13^ found lower passive torque at 30° of ankle dorsiflexion in flexible individuals compared to less flexible individuals (P<0.05), but when comparing the passive peak torque in both groups, flexible participants displayed significantly higher ROM_MAX_ values (P<0.001). Similar outcomes were also reported by Magnusson et al.^17^, who compared knee joint ROM_MAX_ and torque_MAX_ between groups with “normal flexibility” and “reduced ROM”. Conversely, in the present study, torque_MAX_ did not group with the variables ROM_MAX_ and FST_ROM_ in Factor 2 (Factor “sensory”), but it grouped into Factor 1 (Factor “mechanical”) with high factor loading (Table 3). Thus, another interpretation for the torque_MAX_ must be addressed. According to the EFA theory, factors are composed of variables measuring common aspects, and these factors are independent of each other. In this regard, caution must be exercised in assuming that alterations in torque_MAX_ occurred through a modulation of the subjective sensation of stretching in order to explain the increase in ROM_MAX_. Hence, the real nature of the relationship between ROM_MAX_ and torque_MAX_ remains to be clarified.

In summary, the EFA in the present study indicated the existence of a dependency structure of a set of variables described by two major factors. This result supports the viewpoint of Weppler and Magnusson^5^, in which two main theories (mechanical and sensory) have been suggested to describe the MTU response to stretching exercises. Regarding the controversy between the theories to explain acute responses, the present findings support the possibility of a discussion about the structures involved in the MTU adaptation to stretching. However, the use of torque_MAX_ associated with both alterations in individual tolerance to stretching and increases in ROM_MAX_ needs to be further elucidated. Further studies are needed to investigate the long-term effects of stretching using the methods of the present study, and other neurophysiological variables (e.g. the Hoffmann and myotatic reflexes) could also be included to increase the understanding of the structures and mechanisms involved in the MTU adaptation to stretching.
